# PlasmiDB: an open-source and customizable database for plasmid lifecycle management in multi-user, multi-project plant molecular biology laboratories

**DOI:** 10.1186/s13007-026-01555-0

**Published:** 2026-07-01

**Authors:** Alexandre Soriano, Martine Bes, Anne-Cécile Meunier, Celine Georget, Phonsiri Saengram, Sergi Navarro-Sanz, Aurore Vernet, Murielle Portefaix, Thomas Mauran, Maryline Summo, Gaetan Droc, Christophe Périn

**Affiliations:** 1 CIRAD, UMR AGAP Institut, Montpellier, F-34398 France; 2 UMR AGAP Institut, Univ Montpellier, CIRAD, INRAE, Institut Agro, Montpellier, F-34398 France

**Keywords:** Plasmid management, Plant molecular biology, Functional genomics, Plasmid traceability, Open-source database

## Abstract

**Background:**

Functional genomics in plant biology relies on the generation, reuse, and long-term management of large numbers of plasmids produced through diverse cloning strategies. As collections expand across users and projects, laboratories face increasing challenges in organization, traceability, and preservation of construction histories. Existing cloning and sequence-design software supports plasmid design but does not address collaborative, laboratory-wide plasmid management.

**Results:**

We developed PlasmiDB, an open-source, web-based database for managing plasmid collections in multi-user research environments. PlasmiDB provides structured storage of plasmid metadata, explicit tracking of plasmid genealogy, and traceability throughout the plasmid life cycle, including inter-laboratory exchanges. The system implements project-based access control and supports collaborative workflows involving staff, students, and core facilities. Implemented using a standard LAMP architecture and deployable via Docker, PlasmiDB is designed for extensibility without modification of the core database schema. A gene module links genetic targets to associated plasmids, primers, and CRISPR reagents, improving coherence between molecular constructs and experimental objectives. In our laboratory, PlasmiDB currently manages nearly 700 plasmids and facilitates reporting through automated declaration file generation.

**Conclusions:**

PlasmiDB complements existing cloning tools by providing traceability, collaboration support, and long-term data stewardship for plant molecular biology laboratories. The source code and Docker image are publicly available.

**Supplementary Information:**

The online version contains supplementary material available at https://doi.org/10.1186/s13007-026-01555-0.

## Background

Molecular genetics relies on the ability to determine gene function, a goal that supports both fundamental research and numerous applied fields. Achieving this requires the construction and manipulation of large numbers of sequences and plasmids through diverse cloning and engineering strategies. As research laboratories and core facilities produce constructs across multiple users, projects, and funding schemes, plasmid collections rapidly expand and become increasingly heterogeneous [1].

Managing these collections presents several challenges. First, plasmids circulate among researchers, collaborators, and external partners, creating the need for robust traceability, including compliance with institutional policies and material transfer agreements (MTAs). Second, the reconstruction of cloning histories, how a plasmid was derived and from which parent vectors, becomes difficult once constructs multiply or personnel change. Third, the absence of unified, structured storage often leads to redundancy, loss of information, mislabeling, or incomplete documentation, which ultimately slows down research and undermines reproducibility.

Existing tools such as SnapGene software [2], Geneious [3] or Serial Cloner [4] provide efficient solutions for plasmid design, visualization, and sequence manipulation, and in the case of SnapGene even offer a graphical history of cloning operations at the file level. However, these tools are not designed to operate as laboratory-wide plasmid management systems: they do not provide multi-user or multi-project organization, centralized storage, or traceability of plasmid circulation. Their cloning history remains local to individual files and cannot be used to reconstruct or query the genealogy of plasmids across a laboratory collection. PlasmiDB provides a comprehensive multi-user, multi-project system for plasmid tracking and genealogy reconstruction, specifically designed for scientific environments involving multiple collaborations, projects, and heterogeneous organizational constraints (e.g. public/private partnerships, PhD students, and fixed-term researchers).

Open-source inventory systems such as MyLabStocks [5], or synthetic biology registries such as JBEI-ICE [6], offer broader sample management capabilities but do not implement a dedicated model for plasmid–plasmid genealogy and only partially support project-level structuring or external transfer traceability. More broadly, commonly available ELN (Electronic Laboratory Notebook) and LIMS (Laboratory Information Management System) platforms focus on generic sample tracking and therefore do not provide fine-grained plasmid genealogy management. PlasmiDB was designed for academic research environments, offering a flexible framework that allows administrators to adapt data structures (e.g. fields and tags) to evolving needs, unlike conventional LIMS, which are often too rigid for such contexts.

The community resource Addgene serves as a large, publicly accessible plasmid repository for storing and distributing plasmids and associated metadata from thousands of laboratories worldwide [7]. However, Addgene’s database is external and centralised, and cannot be deployed locally or used as a multi-user internal management system for plasmid traceability and project-based organisation like PlasmiDB.

Another important distinction concerns deployment models. Cloud-based platforms offer scalability and ease of access but may raise concerns regarding data control, long-term accessibility, and dependency on external services. In contrast, locally deployable systems provide full control over data and can be integrated within institutional infrastructures, but may require local maintenance and technical setup. Accordingly, PlasmiDB was designed as a locally deployable system to address the specific constraints of institutional laboratory environments. To clarify the positioning of PlasmiDB relative to existing plasmid design and laboratory management systems, a functional comparison is provided in Table 1.

To fill this gap, we present PlasmiDB, an open-source and self-installable database for plasmid management. It supports multi-user and multi-project environments, maintains explicit plasmid genealogy relationships, maintains traceability of plasmid construction throughout the entire plasmid life cycle, including external transfers. Designed for ease of installation and customization, PlasmiDB can be deployed locally in any research laboratory wishing to organise, store, and secure its plasmid collections. PlasmiDB can: (i) store plasmid constructs in a structured, queryable framework, (ii) maintain plasmid genealogy relationships, (iii) ensure intra- and inter-laboratory traceability, and (iv) support collaborative, multi-user, multi-project plasmid management. Moreover, PlasmiDB is freely available and has been designed to be easily extended and customized by the molecular genetics community. Although PlasmiDB was developed in a plant molecular biology context, its architecture is generic and could be adapted to other molecular biology laboratories.

## Construction and content

### Architecture overview and implementation

PlasmiDB is built on a standard LAMP (Linux, Apache, MySQL, PHP) stack. The application follows a MVC (Model–view–controller) architecture. The backend is handled by PHP 8.4, linked to a MySQL 8 database. The user interface is implemented using standard web technologies: HTML5, CSS3, and JavaScript. JavaScript libraries such as DataTables and Select2 are used to make the interface more user-friendly.

PlasmiDB has been deployed in two environments. A Dockerized instance runs alongside other tools on a virtual machine equipped with 12 CPUs and 32 GB of RAM. A legacy non-Docker instance is deployed on a separate virtual machine with 8 CPUs and 16 GB of RAM. Both configurations operate without noticeable performance issues. Our local implementation (“Ariane”) used approximately 512 MB of RAM when deployed with docker.

### Data model and core functions

The database is organized around core entities (plasmids, genes) each defined by multiple associated attributes. These attributes are stored either as free-text fields or as normalized relationships via intermediate tables. The latter uses controlled vocabulary maintained within the database, which is curated by administrators. Plasmids can be created either from scratch or by using another plasmid as a template. Parent-child relationships can be defined, allowing the origin of any plasmid to be traced. It is also possible to define the origin of a plasmid based on multiple source plasmids. This feature is particularly useful when constructing new vectors by assembling sequences derived from several plasmids (e.g., Golden Gate, In-Fusion, Gateway multi-site cloning, etc.). The application also supports the registration of incoming and outgoing plasmid exchanges. This ensures a clear and comprehensive history of each feature’s provenance and subsequent use. Files of different nature can also be associated with these entities. They are stored on disk, within a specific directory tree.

Access control is implemented through a user management system with two authorization levels and project-based permissions. Each plasmid is associated with a project. Administrators have full privileges across the entire database, including user and project permission management, while non-administrator users are granted permissions at the project level. For each project, users can be assigned either read-only or read-write access. For genes, only their creators and administrators are allowed to modify them, except for the primer and spacer entities. In this case, any user can create and view their own primers and spacers for any given gene. An extensive view of the database structure can be found in Supplemental Figure S1.

### User interface

The home page contains a small description of the database and shortcuts to all the main features and functions. This allows users to quickly dive into the specific thing they may want to work with. All the different features in the database can be visualized as global lists, with minor details related to each element, and as detailed pages with all the integrated information. All these lists are sortable and searchable. Administrative functions, like account management or controlled vocabulary modifications, are accessible through an administration panel. Administrators have access to a specific panel. It serves as an access point to all the components of the application. It contains links to all of the system resources, allowing administrators to manage all of them with ease.

### Installation and setup

Installation is supported through Docker Compose, enabling deployment of the application on a server without extensive software configuration. The provided Docker Compose file sets up a fully functional environment including PHP, MySQL, Apache2, and the phpMyAdmin administration interface. Key parameters such as server port mappings and data storage locations can be easily customized through a dedicated configuration file. This facilitates the setup and deployment of one or multiple instances. Database backup scripts are also included and can be scheduled to run periodically, either within the containerized environment or on the host system. User uploaded file persistence is ensured by mounting the upload directory tree on the host system, allowing files to be preserved across deployments and easily backed up. It is also possible to use regular installations of php/mysql to deploy the database using the same variables in the same configuration file.

### SnapGene server

To visualize plasmid maps, users can also deploy the “SnapGene server” software. This tool must be installed separately to enable the related functionalities, and is independent from PlasmiDB. PlasmiDB can communicate with SnapGene server to create on the fly plasmid and gene maps from compatible files format (Serial Cloner, SnapGene, GenBank…). Some SnapGene server helper php functions have been updated to maximize compatibility with the application, and important related variables are accessible and updatable using the global application configuration file.

### Extensibility

The codebase is fully open source and available on a public Git repository (https://gitlab.cirad.fr/alexandre.soriano/plasmidb). Users with PHP expertise can adapt the code to their needs. Its standardized MVC architecture simplifies maintenance and makes the addition of new tables or functionalities more straightforward. PlasmiDB is open-source and distributed under the GNU General Public License v3.0 (GPLv3), allowing users to use, modify, and redistribute the software under the same license.

### SBOL import and export

PlasmiDB supports import and export of plasmid records in the Synthetic Biology Open Language (SBOL) 3 format using the pySBOL3 Python library. Each plasmid can be exported as an SBOL 3 document from its detail page. Standard fields (e.g. plasmid name and description) are mapped to their SBOL equivalents, while associated genes are represented as SBOL features annotated with the Sequence Ontology term SO:0000704 (gene). Metadata not covered by the SBOL 3 specification (e.g. resistances, storage location, project, depositor) are preserved as custom RDF properties within a dedicated namespace. When available, plasmid sequences and annotations extracted from map files are also included in the exported document. SBOL files can be imported through the Add from SBOL entry point. Extracted metadata are used to pre-fill the plasmid creation form, and controlled vocabulary fields are matched against local database entries. Unmatched values are flagged for manual review. When sequence and feature annotations are present, a GenBank map file is automatically reconstructed and linked to the imported plasmid.

### Figures

All figures were created using Biorender.

## Utility and discussion

### Entry page and general navigation

**Fig. 1:**
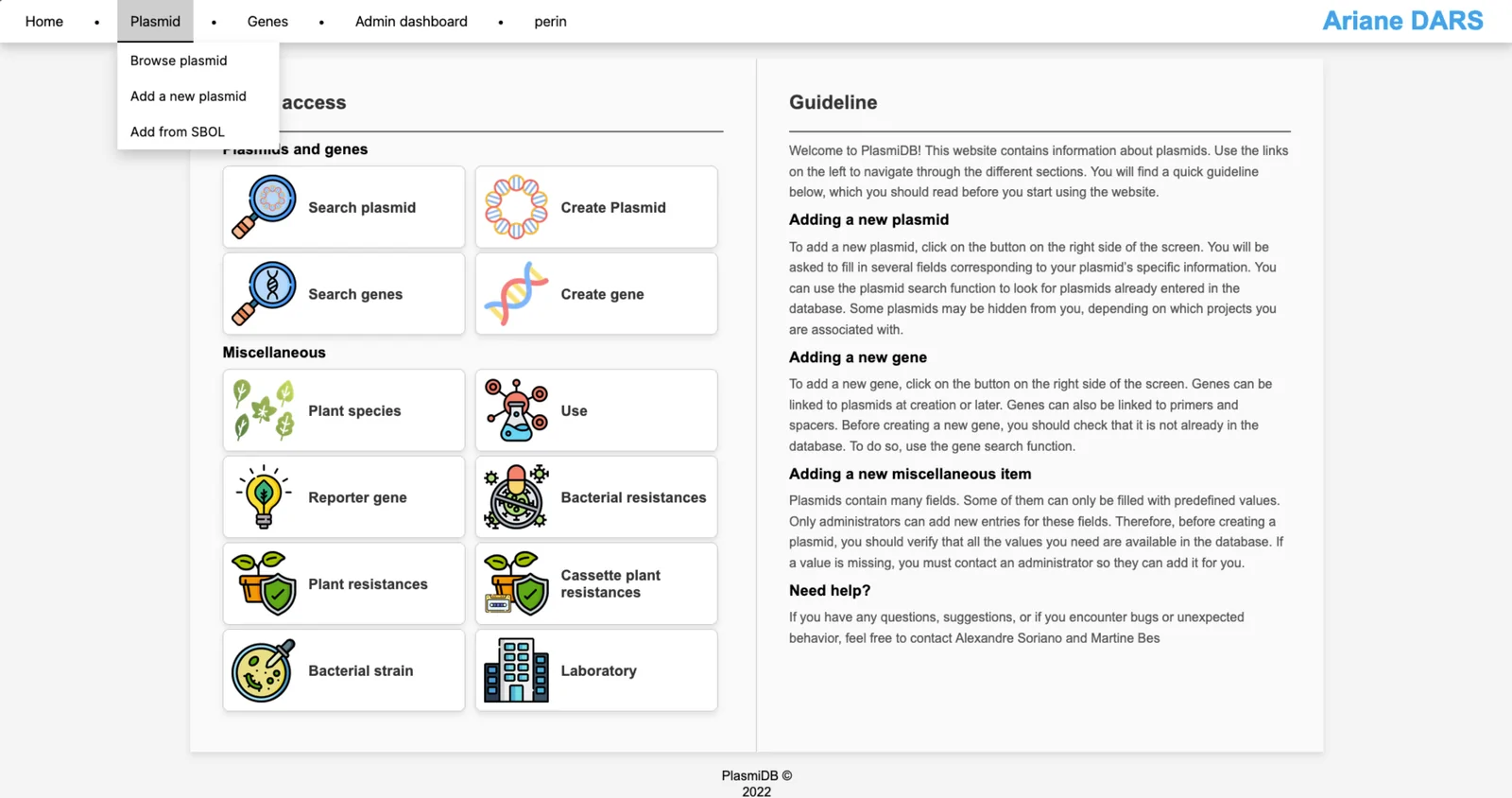
Homepage. The homepage is organized into three main sections. The navigation bar, present on all database pages, provides links and access to the admin dashboard. The admin dashboard allows administrators to manage users and projects. The quick-access area enables users to search or create plasmids, genes, and allows administrators to add generic elements required for managing PlasmiDB (e.g., new antibiotic resistances, new reporter genes). Finally, the guidelines panel offers help and documentation to users. In the upper-right corner of the homepage, the implementation name of PlasmiDB used in our lab, Ariane, is displayed

After logging in, the user is directed to the main entry page (Fig. 1). The interface was designed for clarity and ease of use, with a layout that highlights the most frequently accessed functionalities. All screenshots correspond to a live deployment used in our laboratory. New plasmids can be created either from scratch using the Add New Plasmid option or by importing an SBOL file using the Add from SBOL option, both available from the Plasmid menu (pop up window). The page is structured into three sections. The top bar provides quick access to essential actions, including the administrative dashboard, project and user creation, and general configuration options for PlasmiDB.

Below it, a shortcut panel, ‘Quick access zone’ offers direct links to the core features of the system. This panel includes dedicated entry points for plasmid and gene search or creation, as well as tools for adding auxiliary elements such as plant species, reporter genes, or other components relevant to plasmid construction. Finally, a guideline area summarizes key help resources and provides users with immediate access to documentation and usage instructions.

### User and project management

**Fig. 2:**
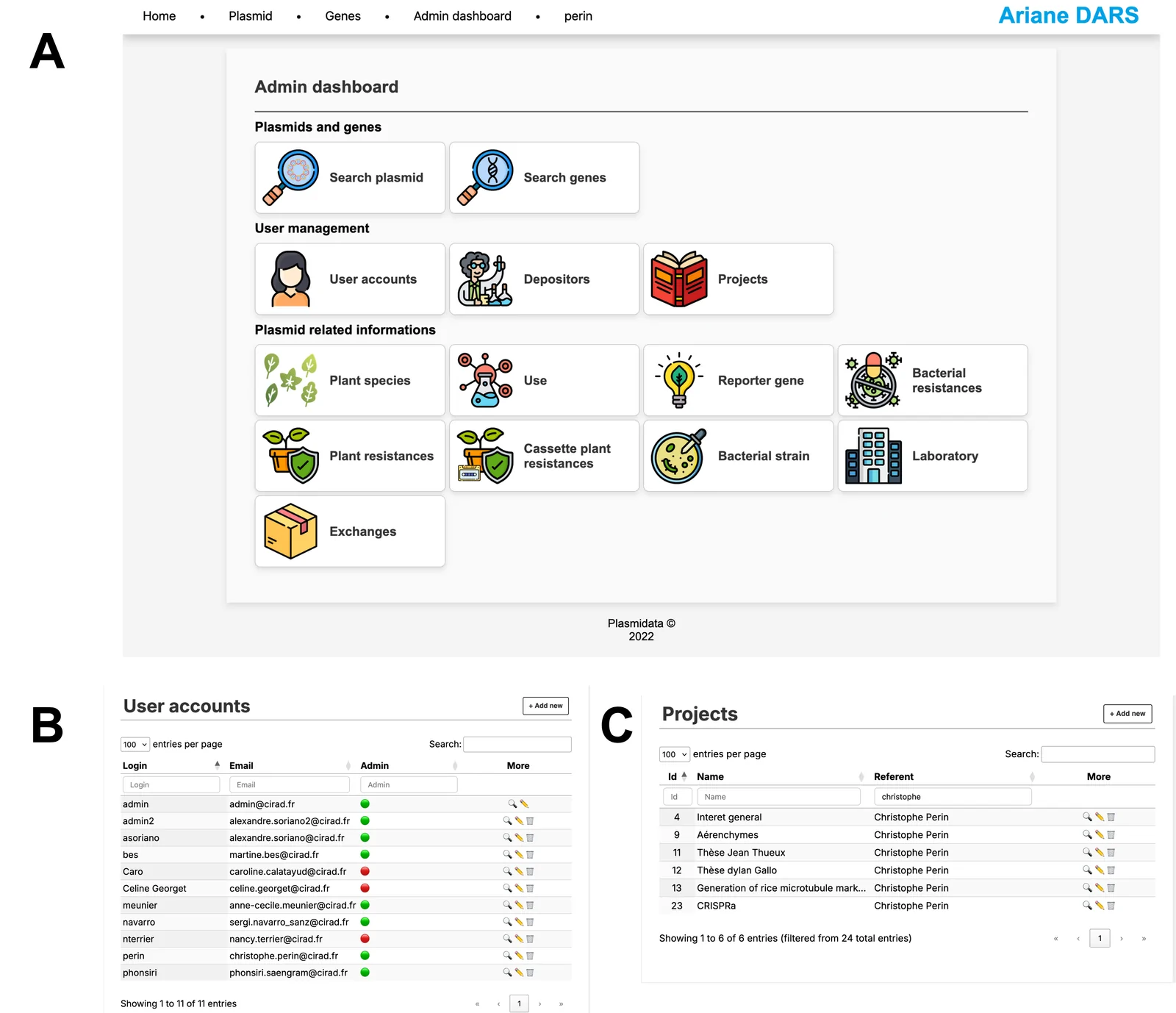
Administrator management interface. Administrator homepage **A** providing links to user management (add, remove, edit users and depositors), project management, and the addition of plasmid-related metadata. These options are also available from the general homepage (see also Fig. 1). User accounts management page **B** listing all registered users along with their information (email, status). A single user may be associated with multiple projects. Project management window **C** displaying all PlasmiDB projects and their designated referents. Administrators can add, edit, or remove projects

The administration dashboard (Fig. 2A) provides access to all features available from the entry page, supplemented with administrator-level functionalities. From this interface, administrators can create or manage user accounts (Fig. 2B), register depositors of genetic materials, and define or update projects stored in the system (Fig. 2C).

### Search plasmids functions and plasmid viewer

**Fig. 3:**
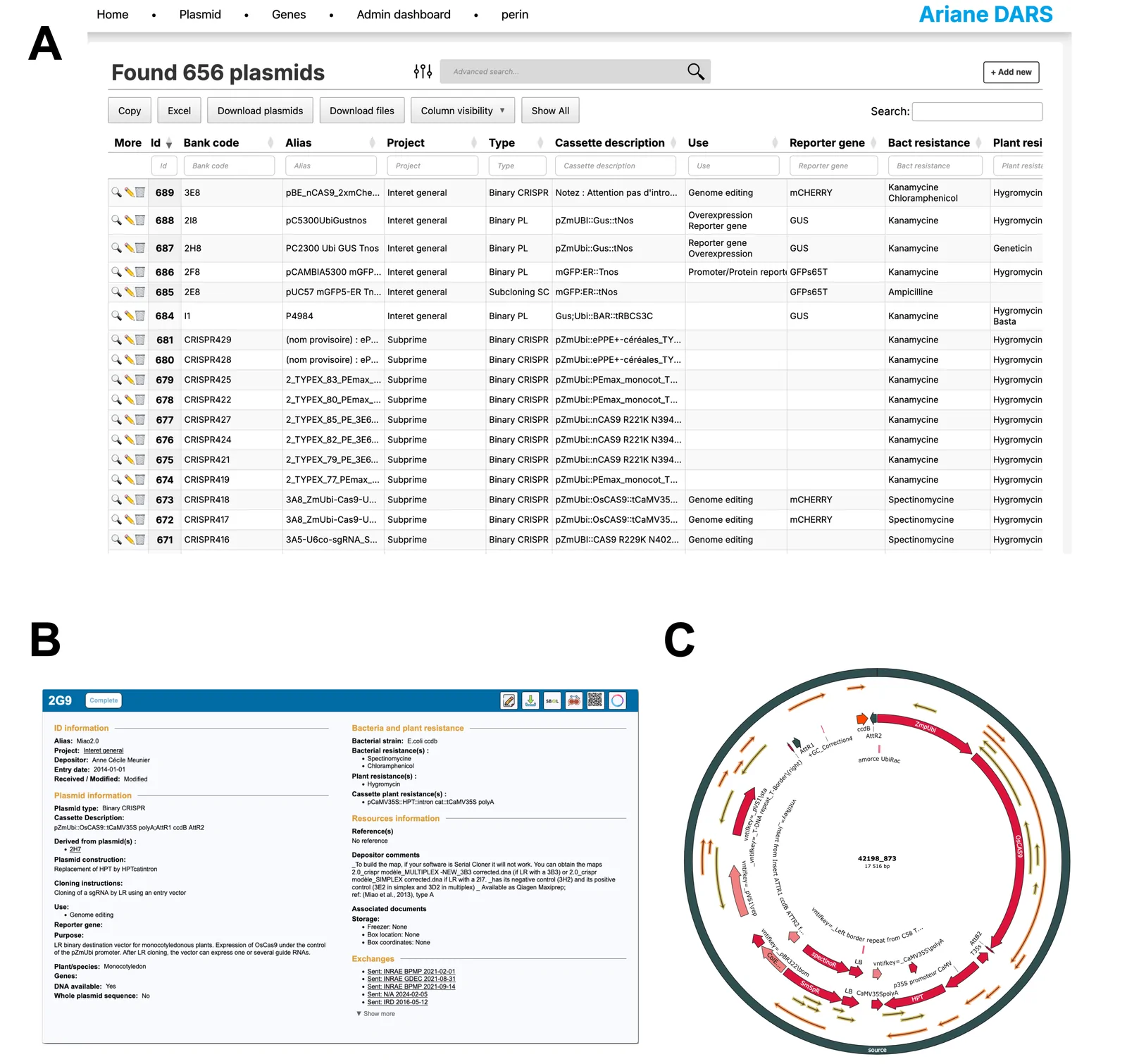
Plasmid search table and plasmid detail view. Plasmids are displayed as rows, with their attributes arranged in columns **A**. Column headers support search, filtering, and sorting. The set of visible columns can be customized using the “Column visibility” button. A global keyword search bar is also available. After applying filters, the complete plasmid list can be exported as a tab-delimited file (Copy button) or as an Excel file (Excel button). In addition, all related information for the filtered plasmids can be downloaded as a flat file (Download file button). For each plasmid, provided the user has the appropriate permissions, its detailed page can be viewed (magnifying-glass icon see **B**), edited (pencil icon), or deleted (trash icon). Plasmid detailed view (**B**). Plasmid-related information can also be exported as an SBOL file using the corresponding button. From left to right, information is organized into sections. The first section groups identifiers and depositor/creator information. The second section contains molecular information, including plasmid type (e.g. binary or cloning vector), cassette description (here, presence of a Cas9 cassette), plasmid construction strategy, and usage guidelines (here, insertion of an sgRNA via LR Gateway cloning). The third section describes the bacterial strain and associated bacterial and plant resistance markers. The last section provides traceability information, including material transfer agreement (MTA), storage location (fridge), and linked publications. Icons on bar, from left to right can be used to edit (pencil), download, duplicate the plasmid; generate a QR code or visualize plasmid map when Snapgene viewer is enabled **C**

The search plasmids page displays plasmid records in a tabular layout, where each row corresponds to a plasmid and each column to one of its attributes (Fig. 3A). In our laboratory implementation (“Ariane”), PlasmiDB currently stores 655 plasmids. At the top of the page, a search bar allows users to query the database using arbitrary word combinations. Below, action buttons enable users to export the filtered plasmid list either as a tab-delimited text file (Copy), an Excel file (Excel), or as downloadable plasmid files (Download plasmids/files). Column visibility can be customised through the Column visibility menu or reset using Show all.

Each plasmid is represented as a single row and can be viewed (magnifying-glass icon), edited (pen icon), or deleted (trash-bin icon), depending on the user’s privileges. Both plasmid visibility and editing rights depend on user roles; only administrators have access to the complete collection. From left to right, information is organized into subsections (See Fig. 3B). The first column groups identifiers and depositor/creator information. The second column contains molecular information, including plasmid type (e.g. binary or cloning vector), cassette description (here, presence of a Cas9 cassette), plasmid construction strategy, and usage guidelines (here, insertion of an sgRNA via LR Gateway cloning). The third column describes the bacterial strain and associated bacterial and plant resistance markers. The last column provides traceability information, including material transfer agreement (MTA), storage location (fridge), and linked publications. A map can be also added and visible through the map button on the plasmid view top bar (Fig. 3C) thanks to the SnapGene web viewer.

### Adding a plasmid

**Fig. 4:**
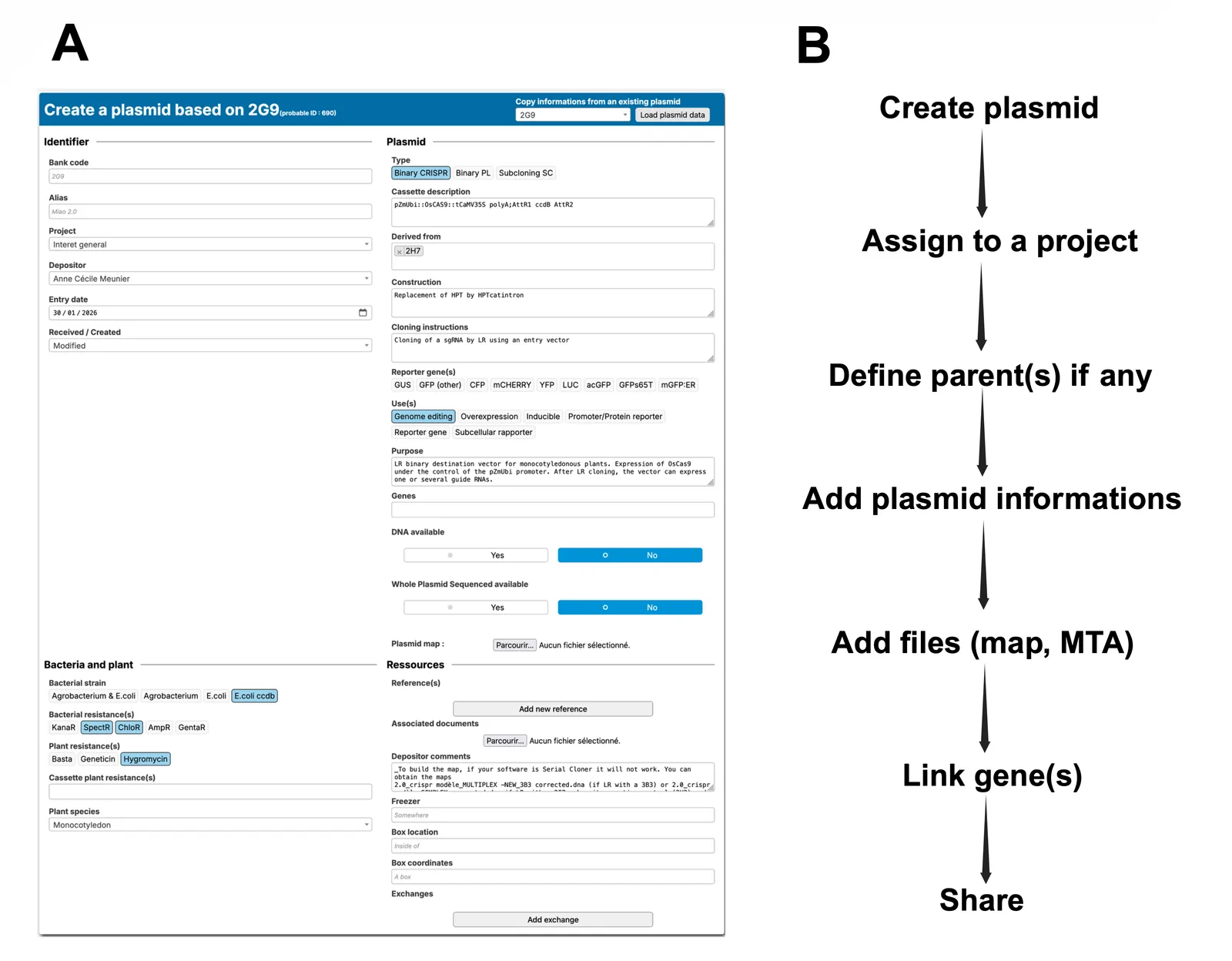
Plasmid creation interface and plasmid creation workflow. The plasmid creation page can be accessed directly from the main page **A**. The top bar allows users to import information from an existing plasmid to accelerate plasmid creation, or alternatively to create a new plasmid from scratch by manual entry. The data fields are identical to those described in Fig. 3. Additional fields can be dynamically added using dedicated buttons, allowing the annotation of multiple features such as bacterial resistance markers or reporter tags (e.g. GFP). Finally, the plasmid map file (.dna, .xdna or .gb format) can be uploaded and downloaded. Any plasmid can be linked to a gene by adding gene names in the corresponding field (see also Fig. 5). Schematic workflow for creating a new plasmid using the plasmid creation interface **B**

The Create new plasmid page (Fig. 4) allows users to register a plasmid either from scratch or as a derivative of existing constructs. When deriving a new plasmid, initial information can be imported directly from a selected backbone already stored in the database. In this case, the copied fields are pre-filled with the source plasmid’s metadata, as illustrated with plasmid 2G9.

The form is organized into four main sections. On the right, the Identifier fields include the internal bank code, used to reference the physical DNA sample, and an alias corresponding to the user-defined name. The Plasmid section gathers general information about the construct, including its derivation from one or several existing plasmids and any relevant usage notes or cloning instructions. The Genes field is used when the plasmid carries gene-related modules already present in the database, for example constructs designed for knock-out, promoter fusions or other functional analyses. The Bacterial and Plant sections collect information on host species, selectable markers, strains, and other organism-specific attributes. The final section centralizes traceability elements such as publications, MTAs and exchange records.

### Gene module

**Fig. 5:**
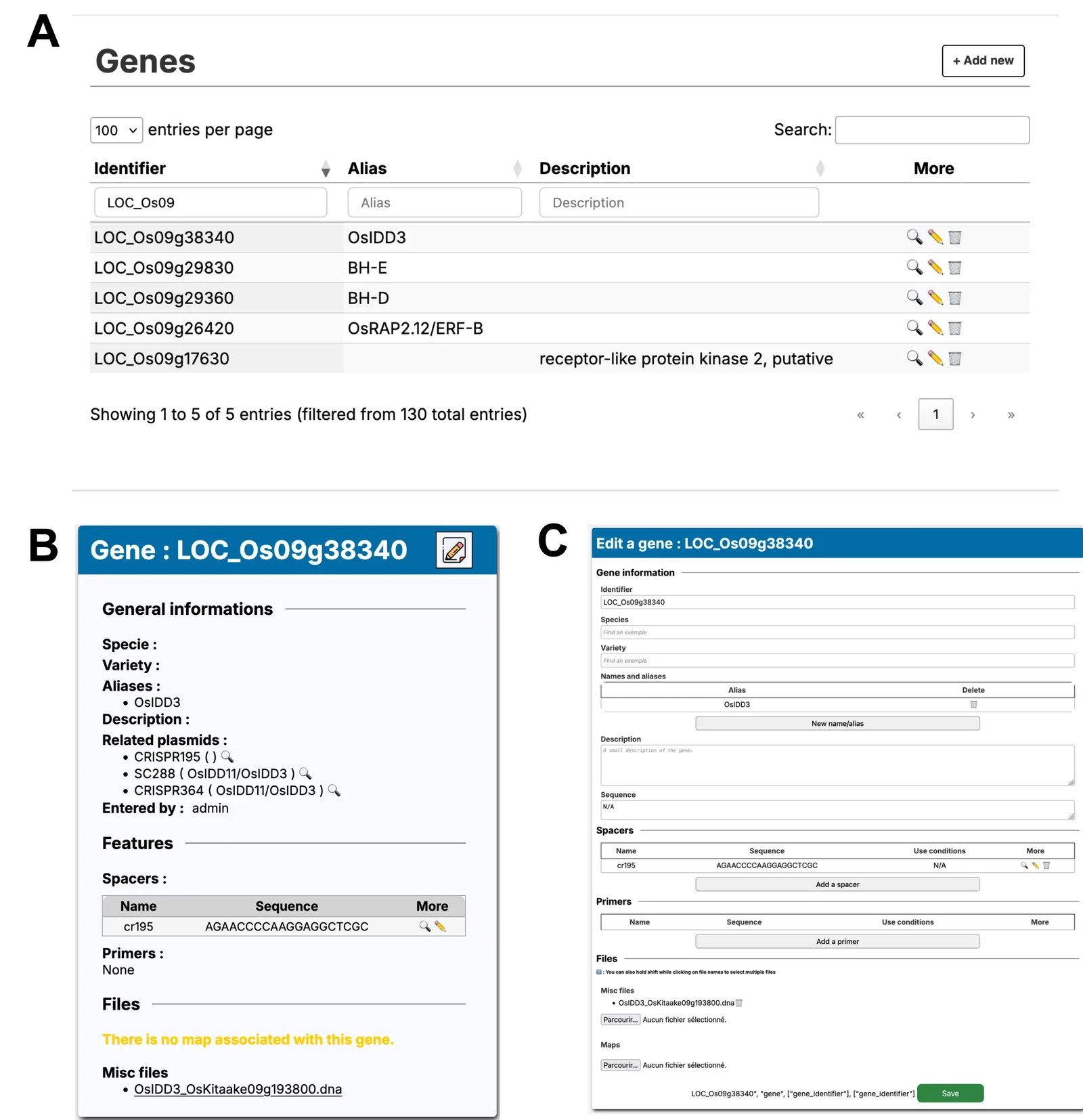
Gene search, visualization, and creation interfaces. Users can search the gene list **A** using column-based filters or a global search field, and access any gene stored in the database. For each gene, provided the user has appropriate permissions, the detailed gene page can be viewed (magnifying-glass icon; **B**), edited (pencil icon), or deleted (trash icon). **B** displays the detailed information page for gene LOC_Os09g38340. In addition to gene attributes, including a genomic map, available CRISPR spacers, and primers (when available), users can access all plasmids developed for functional analysis of the gene. In this example, three plasmids corresponding to single or double knock-out lines are shown. The “Add new” button allows the creation of a new gene entry. A gene can be edited also by clicking on a pencil-like icon (see **C**). The gene module of the database was designed to facilitate efficient retrieval of gene-related information and associated plasmids developed for functional analyses

PlasmiDB database was initially designed to facilitate plasmid construction, sharing, and development for functional analyses. To this end, we implemented a dedicated module to store and manage gene-related information. Genes can be searched and sorted in a manner similar to plasmids, using a global search function or column-based filtering (Fig. 5A). By clicking on the magnifying-glass icon, users can access the detailed view of a gene (Fig. 5B), illustrated here with LOC_Os09g38340. In this example, three plasmids were developed for functional analysis of LOC_Os09g38340 using CRISPR/Cas9. Primers, guide RNA spacers, and additional plasmids (e.g. promoter–gene fusions or overexpression constructs) can also be associated with the gene entry. Gene map, when available, is accessible thanks to the SnapGene web viewer. Finally, new gene entries can be created using the “Add new” button, and existing entries can be edited via the pencil icon (Fig. 5C).

### Sharing and working together: PlasmiDB in real life

**Fig. 6:**
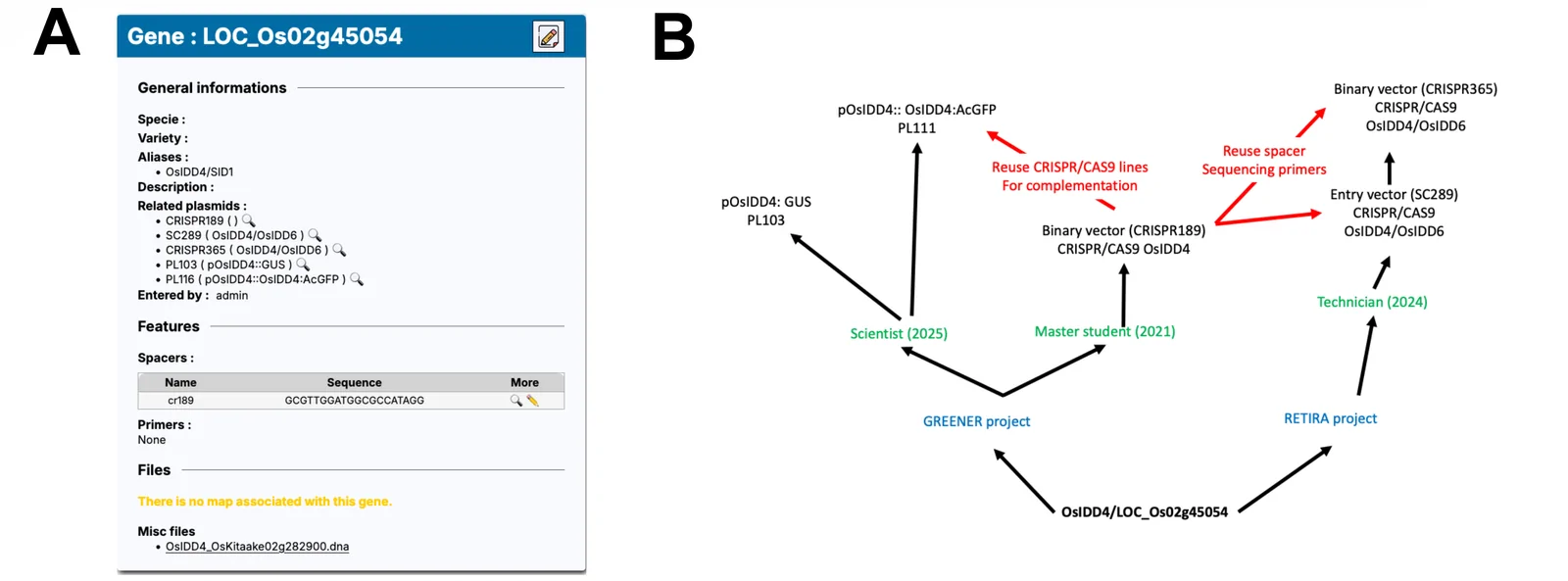
Example of collaborative plasmid management using PlasmiDB. Gene visualization of plasmids constructed for the functional analysis of the rice gene OsIDD4 across multiple projects **A**. Five plasmids were generated within distinct research contexts, including the GREENER ANR project and the RETIRA project, involving different contributors (student, technician, researcher). Pasmid genealogy and reuse of genetic elements in PlasmiDB for OsIDD4 **B**. A CRISPR spacer initially designed by a Master’s student (SC208) was subsequently reused in multiplex CRISPR/Cas9 constructs targeting OsIDD genes (e.g. SC289, CRISPR365) developed in another project. PlasmiDB allows tracking of relationships between constructs and provides centralized access to plasmid sequences, maps, and associated experimental information. For example, a translational fusion construct (PL111) can be readily identified and retrieved to complement OsIDD4 CRISPR/Cas9 mutant lines generated using CRISPR365. Projects are in blue, users in green, reuse of constructs in red

In this example, the functional analysis of the rice gene OsIDD4 involves multiple constructs developed across different projects. Five plasmids were constructed for the functional analysis of OsIDD4 (Fig. 6A). The first construct was a single CRISPR design generated within the GREENER project by a Master’s student (SC208) (Fig. 6B). Subsequently, multiplex CRISPR constructs targeting several OsIDD genes, including OsIDD4 (e.g. OsIDD4/OsIDD6, SC289; CRISPR365), were developed within the RETIRA project by a technician. Finally, additional constructs, including transcriptional (PL103) and translational fusions (PL111), were generated by a researcher within the ANR framework.

This example illustrates the importance of a shared plasmid management system such as PlasmiDB. First, previously designed elements (e.g. CRISPR spacers generated by the Master’s student) can be directly reused in subsequent constructs developed by other team members. In this example, a spacer was reused in multiplex CRISPR/Cas9 constructs (Fig. 6B). Second, all constructs, gene maps, and associated sequence information are centrally accessible, allowing users to retrieve key experimental details such as sequencing primers, spacer sequences, and their genomic positions.

More broadly, PlasmiDB facilitates the sharing of critical technical information that is otherwise fragmented across individual computers, notebooks, or storage systems. It enables efficient reuse of constructs and components, reduces redundancy, and improves continuity between projects and personnel. For instance, a researcher aiming to complement an OsIDD4 CRISPR/Cas9 mutant can directly identify and retrieve the appropriate clone, in this case PL111, from PlasmiDB (Fig. 6B) to complement knock-out mutant lines developed with CRISPR365.

## Conclusion

PlasmiDB was initially developed to address practical challenges encountered in everyday laboratory work, namely plasmid traceability, storage, and collaborative use in multi-user environments. In its current internal deployment, the database already manages nearly 700 plasmids, illustrating its suitability for medium to large-scale plasmid collections. In our current deployment, PlasmiDB supports 20 users across 20 projects without noticeable performance issues during routine laboratory use. By providing a centralized, structured, and searchable repository, PlasmiDB simplifies access to plasmid information across projects, teams, and user profiles, thereby reducing redundancy, information loss, and inefficiencies linked to fragmented data storage.

PlasmiDB is not intended to replace cloning or sequence-design software such as SnapGene, whose primary purpose is plasmid construction and sequence manipulation. Instead, it complements these tools by focusing on plasmid lifecycle management in complex and heterogeneous working environments, including shared platforms, research teams, permanent staff, and students. By integrating plasmid genealogy, traceability, project-based access control, and external exchange records, PlasmiDB provides a long-term solution for organizing plasmid resources beyond individual projects or personnel turnover.

A key strength of PlasmiDB lies in its flexibility and extensibility. The database is open-source and can be easily customized to meet the specific needs of individual laboratories. Administrators can extend controlled vocabularies, for example by adding new selectable markers or reporter tags, without modifying the underlying database schema. Moreover, the PHP-based architecture allows experienced users to adapt existing tables or introduce new ones with limited development effort, making PlasmiDB suitable for evolving experimental strategies and technological advances such as new fluorochromes, selectable marker genes, and emerging cloning and genome editing technologies.

Beyond plasmid management, the optional gene module further enhances the utility of PlasmiDB by linking genetic targets to associated constructs, primers, and CRISPR reagents. This integration facilitates functional genomics workflows by enabling rapid retrieval of all resources developed for a given gene, thereby improving coherence between molecular design, experimental execution, and long-term data stewardship.

General-purpose ELN and LIMS systems treat plasmids as standard laboratory samples within a broad inventory framework [8]. However, they typically lack explicit mechanisms to reconstruct plasmid–plasmid genealogy or to manage multi-source vector assembly histories. PlasmiDB introduces a plasmid-specific relational model that captures construction lineage, parent–child relationships, and inter-project circulation in a structured and queryable manner. This plasmid-centric design addresses needs that extend beyond generic sample management.

In summary, PlasmiDB provides a lightweight yet robust solution for plasmid management in molecular biology laboratories, particularly in plant research contexts. By emphasizing traceability, collaboration, and extensibility, it addresses a gap between plasmid design software and generic inventory systems. We anticipate that PlasmiDB will be a valuable resource for laboratories seeking to organize, secure, and sustain their plasmid collections over time, and we hope that its open-source nature will encourage adoption, adaptation, and further development by the research community.

PlasmiDB now supports import and export of plasmid records in the SBOL 3 (Synthetic Biology Open Language) format, improving interoperability with external resources. Future developments could further extend SBOL support and integration with community platforms such as SynBioHub. Interoperability with external plasmid repositories and databases represents also an important perspective for PlasmiDB. The system allows users to store external identifiers (e.g. Addgene IDs, publication references) and associated metadata. Its flexible data structure also enables the addition of custom fields to facilitate links with external resources. Future developments could include the implementation of APIs and standardized data formats to improve interoperability with public repositories and other laboratory information systems. We anticipate that these future developments could benefit from interactions with the broader synthetic biology community.

**Table 1:**
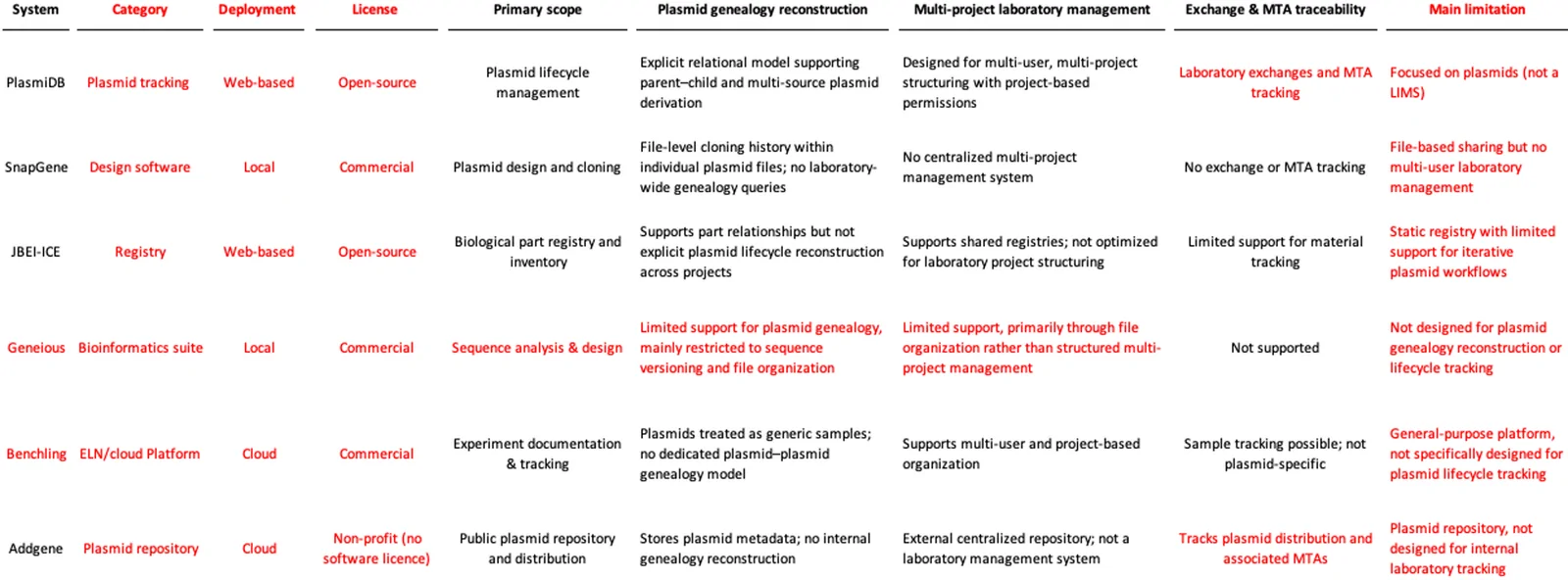
Functional comparison of PlasmiDB with representative plasmid design and laboratory management systems

## Supplementary Material

Supplementary material 1.

## Data Availability

The source code of PlasmiDB and a Docker image for deployment are available at (https://gitlab.cirad.fr/alexandre.soriano/plasmidb). All documentation and example configuration files are provided in the repository. Additional data supporting the findings of this study are included in the article and its supplementary material.

## References

[1] Tian C, Li J, Wu Y, Wang G, Zhang Y, Zhang X, Sun Y, Wang Y. An integrative database and its application for plant synthetic biology research. Plant Commun. 2024;5(5):100827. 38297840 10.1016/j.xplc.2024.100827 PMC11121754

[2] SnapGene. SnapGene software. Available from: www.snapgene.com. Accessed 2026 Feb 12.

[3] Kearse M, Moir R, Wilson A, Stones-Havas S, Cheung M, Sturrock S, Buxton S, Cooper A, Markowitz S, Duran C, et al. Geneious Basic: an integrated and extendable desktop software platform for the organization and analysis of sequence data. Bioinformatics. 2012;28(12):1647–9. 22543367 10.1093/bioinformatics/bts199 PMC3371832

[4] Serial cloner. Serial Cloner software. Available from: http://serialbasics.free.fr/Serial_Cloner.html. Accessed 2026 Feb 12.

[5] Chuffart F, Yvert G. MyLabStocks: a web-application to manage molecular biology materials. Yeast. 2014;31(5):179–84. 24643870 10.1002/yea.3008 PMC4019915

[6] Ham TS, Dmytriv Z, Plahar H, Chen J, Hillson NJ, Keasling JD. Design, implementation and practice of JBEI-ICE: an open source biological part registry platform and tools. Nucleic Acids Res. 2012;40(18):e141. 22718978 10.1093/nar/gks531 PMC3467034

[7] Kamens J. The Addgene repository: an international nonprofit plasmid and data resource. Nucleic Acids Res. 2015;43(Database issue):D1152–1157. 25392412 10.1093/nar/gku893 PMC4384007

[8] Labguru. Labguru electronic laboratory notebook and LIMS platform. Available from: https://www.labguru.com. Accessed 2026 Feb 12.

